# A genetic determinant of VEGF-A levels is associated with telomere attrition

**DOI:** 10.18632/aging.203636

**Published:** 2021-10-18

**Authors:** Vesna Gorenjak, Alexandros M. Petrelis, Maria G. Stathopoulou, Simon Toupance, Satish Kumar, Carlos Labat, Christine Masson, Helena Murray, John Lamont, Peter Fitzgerald, Athanase Benetos, Sophie Visvikis-Siest, TELARTA Consortium

**Affiliations:** 1 Université de Lorraine, IGE-PCV, Nancy F-54000, France; 2 Université de Lorraine, Inserm, DCAC, Nancy F-54000, France; 3 Randox Laboratories Limited, Crumlin, Co. Antrim BT29 4QY, Northern Ireland, United Kingdom; 4 Université de Lorraine, CHRU-Nancy, Pôle “Maladies du Vieillissement, Gérontologie et Soins Palliatifs”, Nancy F-54000, France

**Keywords:** VEGF-A, telomere length, telomere attrition, accelerated cellular aging

## Abstract

Telomere length (TL) is a hallmark of cellular aging and is associated with chronic diseases development. The vascular endothelial growth factor A (VEGF-A), a potent angiogenesis factor, is implicated in the pathophysiology of many chronic diseases. The aim of the present study was to investigate the associations between VEGF-A and TL.

TL in leukocytes (LTL) and skeletal muscle (MTL) were measured, 10 VEGF-related polymorphisms genotyped, and VEGF-A plasma concentrations determined in 402 individuals from the TELARTA cohort. LTL/MTL ratio was calculated as an estimate of lifelong TL attrition. Associations between VEGF-A variants and levels, and TL parameters were investigated.

We identified one significant association between the minor allele (T) of rs6993770 variant and LTL/MTL ratio (P=0.001143, β=0.0148, SE=0.004516). The rs6993770 is an intronic variant of the *ZFPM2* gene, which is involved in haematopoiesis and the identified association with increased telomere attrition could be due to increased haematopoiesis. No significant epistatic interaction was identified, and no association was found between levels of VEGF-A and any of assessed phenotypes.

We identified a potential common genetic regulation between VEGF-A and telomere length attrition that could be explained by mechanisms of increased hematopoiesis and production of platelets. VEGF-A and TL could play an important role in personalized medicine of chronic diseases and identification of molecular links between them can promote the understanding of their complex implications.

## INTRODUCTION

Telomeres are repeated sequences of TTAGGG nucleotides at the end of eukaryotic chromosomes, participating in the maintenance of chromosomal integrity during cell replication [1]. As telomere length (TL) is gradually shortening with each mitotic division it has been considered as an indicator of accelerated cellular aging. Inflammation and oxidative stress are increasing the shortening rate of TL and further affect the aging process and are considered as a basic link between TL and many chronic diseases [2]. Short leukocyte TL (LTL) is associated with the pathogenesis of atherosclerosis [3]. This process is characterised by increased inflammation, mediated by a production of cytokines mainly from blood-borne inflammatory and immune cells, but also from vascular endothelial and smooth muscle cells [4]. TL has also been associated with other chronic diseases such as cancer [5], osteoporosis [6], chronic kidney diseases [7], neurodegenerative diseases [8] and others.

LTL is known for its high interindividual variation, which is evident already at birth [9]. Afterwards, numerous factors may accelerate LTL attrition, e.g. increased cellular turnover caused by increased inflammation, oxidative stress [10], or may have a protective effect on LTL shortening, e.g. regular sport activity, healthy diet [11]. Although the effects of these factors on LTL are rather small compared to the large variation observed in the baseline TL among individuals [12], the use of LTL alone as a biomarker could be not as informative as it would be in combination with an estimate of lifelong telomere attrition [13]. As skeletal muscle is a minimally proliferative tissue, skeletal muscle TL (MTL) can be considered as a baseline TL proxy, and estimated lifelong TL attrition can be calculated by the LTL/MTL ratio [14].

The vascular endothelial growth factor A (VEGF-A) is a key regulator of physiological and pathological angiogenesis and a mediator of vascular permeability in inflammatory disorders [15]. It plays a critical role in cancer and several VEGF-A signaling inhibitors have already been approved by the Food and Drug Administration (FDA) for oncological treatment [16]. VEGF-A also seems to be important in cardiovascular diseases (CVD) [17], including atherosclerosis, where it promotes neoangiogenesis in the early stages of the disease as well as in the advanced atherosclerotic plaques [18].

VEGF-A and TL are both involved in chronic diseases pathways, such as inflammation and angiogenesis. They have been commonly assessed in studies of different conditions [19–22], however, their associations have not been extensively studied [10, 23]. Furthermore, these biomarkers are highly heritable [24, 25] and have an important inter-individual variability [26, 27] and could have a great value in personalized medicine as they could become a new tool for calculation of risk factors in personalized diagnostics of chronic diseases. The identification of common genetic determinants for both markers could play an important role towards this direction. Therefore, in order to expand the knowledge of the common pathways that regulate both biomarkers, the current study aimed to investigate the genetic associations between VEGF-A and TL.

Accordingly, ten variants that were associated in a GWAS study with VEGF-A levels and together explain up to 52% of the VEGF-A phenotypic variance [28] were investigated for association with LTL and MTL, as well as estimated lifelong telomere attrition (LTL/MTL ratio) in a cohort of adult subjects.

## RESULTS

The descriptive characteristics of the population are presented in Table 1.

**Table 1:** Characteristics of the population.

**Variable**	**n**	**mean**	**SD**
Age (years)	402	60	15
Sex (F/M %)	402	32/68	
LTL (kb)	402	6.71	0.84
MTL (kb)	402	8.57	0.72
LTL/MTL	402	0.78	0.07
VEGF-A (pg/ml)	324	25.55	16.97
Statin use (%)	324	38.88	

SD, standard deviation.

LTL and VEGF are not normally distributed so median values and interquartile ranges are also given.

LTL: 6.62 (6.11 – 7.26) kb.

VEGF-A: 15.79 (10.07 - 29.38) pg/ml.

### Association between VEGF-A related genetic variants and TL

Values of MAF and results of HWE analysis are presented in Table 2.

**Table 2:** Results of MAF and HWE analysis for ten VEGF-A related genetic variants in the study population.

**SNP**	**Chromosome**	**MAF**	**HWE (P-value)**
rs114694170	5	0.04602	0.5802
rs34528081	6	0.3505	0.3788
rs6921438	6	0.4325	1
rs4416670	6	0.4527	0.4209
rs6993770	8	0.2873	1
rs7043199	9	0.206	0.7591
rs10738760	9	0.4739	0.617
rs10761741	10	0.4439	0.01131*
rs4782371	16	0.3282	0.1659
rs2639990	18	0.1136	0.6178

MAF, Minor allele frequency; HWE, Hardy-Weinberg equilibrium; *significant P-value.

The SNP rs10761741 did not follow the HWE and was excluded from further analyses.

None of the 9 assessed SNPs was significantly associated with LTL (Table 3) or MTL (Table 4).

**Table 3:** Association between VEGF-A related genetic variants and LTL (log transformed)*.

**SNP**	**Chromosome**	**β**	**SE**	**P-value**
rs114694170	5	0.002083	0.007452	0.7799
rs34528081	6	-0.001541	0.003261	0.6369
rs6921438	6	-0.0004508	0.003173	0.8871
rs4416670	6	-0.002907	0.00323	0.3687
rs6993770	8	0.007	0.003496	0.04594
rs7043199	9	-0.00173	0.003846	0.6531
rs10738760	9	0.002185	0.003119	0.4841
rs4782371	16	0.002397	0.003291	0.4669
rs2639990	18	-0.001899	0.004965	0.7023

B, Effect size; SE, Standard error; P-value threshold is P<0.0012.

*models are adjusted for age and sex.

**Table 4:** Association between VEGF-A related genetic variants and MTL*.

**SNP**	**Chromosome**	**β**	**SE**	**P-value**
rs114694170	5	0.1713	0.1132	0.1312
rs34528081	6	-0.07143	0.04946	0.1495
rs6921438	6	-0.06758	0.04827	0.1623
rs4416670	6	0.007034	0.04927	0.8866
rs6993770	8	-0.02124	0.05354	0.6917
rs7043199	9	-0.08772	0.05874	0.1361
rs10738760	9	0.03656	0.04753	0.4422
rs4782371	16	0.02819	0.05035	0.5758
rs2639990	18	0.06804	0.07592	0.3707

B, Effect size; SE, Standard error; P-value threshold is P<0.0012.

*models are adjusted for age and sex.

A significant association was identified between the minor allele (T) of rs6993770 and LTL/MTL (P=0.001143, β=0.0148, SE=0.004516) (Table 5). There was no significant epistatic interaction identified for three tested phenotypes (LTL, MTL and LTL/MTL) according to the selected threshold (p<0.00046).

**Table 5:** Association between VEGF-A related genetic variants and LTL/MTL*.

**SNP**	**Chromosome**	**β**	**SE**	**P-value**
rs114694170	5	-0.01288	0.009716	0.1856
rs34528081	6	0.004154	0.004246	0.3285
rs6921438	6	0.004166	0.004146	0.3156
rs4416670	6	-0.005934	0.004207	0.1592
rs6993770	8	0.0148	0.004516	0.001143*
rs7043199	9	0.004878	0.005062	0.3358
rs10738760	9	0.0003862	0.004077	0.9246
rs4782371	16	0.002534	0.004284	0.5546
rs2639990	18	-0.008642	0.006481	0.1832

SE, Standard error; β, Effect size; P-value threshold is P<0.0012, *significant P-value.

*models are adjusted for age and sex.

**Figure 1:**
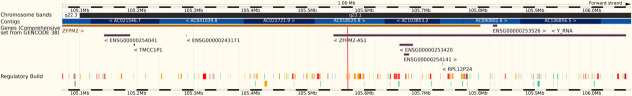
Rs6993770 (red stripe) is located on the intron 4 of the *ZFPM2* gene (8q23.1).

A bioinformatics analysis showed that rs6993770 is located on the intron region (Figure 1) of the *ZFPM2* (zinc finger protein, FOG family member 2) gene.

### Association between VEGF-A plasma concentrations and TL

VEGF-A plasma concentrations were tested for association with LTL, MTL and LTL/MTL using multiple regression analysis (Table 6) with VEGF-A levels used as the dependent variable. There was no significant relation of VEGF-A plasma concentrations with the investigated TL phenotypes.

**Table 6:** Univariate and multiple regression analysis of VEGF-A plasma concentration with LTL, MTL and LTL/MTL.

Independent variable*	**Regression coefficient (SE)**	**R^2^**	**P-value**
**Non adjusted**			
LTL	0.019 (0.160)	0.19%	0.42
MTL	-0.009 (0.028)	0.03%	0.74
LTL/MTL	0.471 (0.300)	0.73%	0.12
Adjusted*			
LTL	-0.075 (0.107)	0.15%	0.49
MTL	-0.170 (0.105)	0.81%	0.36
LTL/MTL	0.756 (0.919)	0.21%	0.41

SE, Standard error.

*each TL variable was tested as independent variable in separate models. Thus, the table presents the results of 3 different models. * Models were adjusted for age, sex and statin use.

Dependent variable: logVEGF-A.

## DISCUSSION

The present study investigated associations between VEGF-A genetic determinants and telomere length dynamics. It identified a direct association between minor allele (T) of rs6993770 and LTL/MTL ratio (P=0.001143). This result suggests a common genetic regulation between VEGF-A and telomere attrition, possibly through a molecular process that affects both biomarkers.

Rs6993770 is one of the most significant variants associated with circulating VEGF-A levels. The minor allele (T) of SNP has been previously related to decreased VEGF-A levels. Together with three other SNPs (rs6921438, rs4416670 and rs10738760), rs6993770 explained 48% of the heritability of serum VEGF-A levels [29]. Besides VEGF-A, it has been related to variation in HDL cholesterol [30], erythrocyte count, IL-12 levels, and platelets count [31].

In the blood, VEGF-A can be found in plasma, platelets and leukocytes [32]. Several studies reported the correlation between the concentration of VEGF-A and platelets, which are particularly important in wound healing and may have a stimulating role in angiogenesis-dependent tumor growth through their function as transporters of VEGF-A [33, 34].

In the present study, the minor allele (T) of rs6993770 was associated with increased LTL/MTL ratio, indicating the protective role of allele (T) in telomere attrition. Rs6993770 is located in the intron of the *ZFPM2* gene, coding for a FOG (Friend of GATA) family member protein. The FOG proteins can both activate and down-regulate expression of GATA-target genes, resulting in modulation of GATA family proteins activity. The *ZFPM2* gene codes for the FOG family member 2 that has been linked with repression of GATA mediated transcriptional activation [35, 36]. GATA proteins are crucial regulators of haematopoiesis and cardiogenesis *via* the control of haemoglobin synthesis [37].

The genetic variant rs6993770 could likely impact on the activity of the *ZFPM2* gene, which would, in turn, affect GATA protein regulation of haematopoiesis. The risk allele (A) of the identified SNP could lead to increased haematopoiesis, which may result in high cellular turnover and thus, faster telomere attrition [38]. Moreover, increased haematopoiesis could lead to bigger production of platelets, which would explain the higher levels of VEGF-A in subjects with this risk variant and the previously identified association of rs6993770 with platelets [39]. Such a hypothesis seems plausible since telomere attrition was the only TL phenotype that was significantly related to the genetic variant, whereas LTL and MTL did not show significant association with rs6993770. Estimated lifelong attrition as expressed by the LTL/MTL ratio has been suggested to be impacted mainly by TL attrition during early life [40]. The finding of our study could be in agreement with this statement. The risk allele (A) of SNP rs6993770 may cause increased haematopoiesis and thus increased leukocyte telomere attrition, especially in childhood, when cellular turnover is the highest.

Besides the genetic association between TL and VEGF-A, this study did not identify any direct association between VEGF-A plasma levels and LTL, MTL or estimated telomere attrition. The previous studies which investigated the association between LTL and VEGF-A levels reported inconsistent findings. No statistically significant association was reported between LTL and plasma concentrations of VEGF-A in the longitudinal study population consisting of 87 subjects [41]. On the other hand, a study of patients with knee osteoarthritis identified a negative correlation between VEGF-A plasma levels and LTL [42]. Further studies are warranted to fully explore the association of these biomarkers. Thus, the genetic association of rs6993770 with telomere attrition seems to not be directly linked with VEGF-A levels and is probably mediated by other mechanisms, such as the increased haematopoiesis that we propose here.

We would like to acknowledge the limitations of the study. The power is limited due to small sample size, so false negative results cannot be excluded. Therefore, we propose the replication of the results in bigger populations.

To conclude, this study is the first to investigate the association of VEGF-A related genetic variants with LTL, MTL and telomere attrition. The study identified a significant association between rs6993770 and telomere attrition. We propose that this association could be explained possibly through the modification of the expression of GATA proteins, which could result in a direct impact on hematopoiesis and production of platelets. This hypothesis remains to be replicated and verified in future studies.

## MATERIALS AND METHODS

### Population

This analysis included 402 individuals with measurements of LTL and MTL from the TELARTA (TELomere in ARTerial Aging) cohort. The aim of the TELARTA study was to examine the role of telomere length dynamics in arterial aging using the “Blood-and-Muscle model” [43, 44]. Briefly, 259 French individuals, who were admitted for various surgical procedures, were recruited in two centers (Nancy and Marseille) and constituted the discovery cohort of the TELARTA study. The replication cohort of the study included 91 French individuals, recruited under the same conditions as in the discovery cohort and 52 individuals from an independent Greek population enrolled in Athens. All French participants provided written informed consent approved by the Ethics Committee (Comité de Protection des Personnes) of Nancy, France. All Greek participants provided written informed consent approved by the Ethics Committee of the University of Athens and Ethics Committee of each one of the three participating hospitals. The study was conducted in accordance with the Declaration of Helsinki and is registered on http://www.clinicaltrials.gov under unique identifier: NCT02176941.

### Telomere length measurement

TL in skeletal muscle (MTL) and in leukocytes (LTL) were measured in DNA extracted from muscle biopsies and peripheral blood leukocytes respectively [14, 43]. Skeletal muscle biopsies (~100 to 200 mg in the surgical field) were collected from individual during surgery, flash frozen in liquid nitrogen and stored on -80° C until DNA extraction. Whole blood samples were collected in EDTA tubes prior to surgery and stored on -80° C until DNA extraction.

DNA was extracted from the muscle tissue and leukocyte by the phenol/chloroform/isoamyl alcohol method. DNA samples passed an integrity testing using a 1% (wt/vol) agarose gel before TL measurement was performed by the Southern blot analysis of terminal restriction fragments, as described previously [45]. Briefly, DNA samples were treated overnight with restriction enzymes *Hinf*I and *Rsa*I (Roche Diagnostics GmbH, Germany). Digested DNA samples and DNA ladder were resolved on 0.5% (wt/vol) agarose gels for 23 hours. After depurination, denaturation and neutralization, DNA was transferred on a positively charged nylon membrane (Roche) using a vacuum blotter (Biorad, Hercules, CA, USA). Membranes were hybridized at 42° C with a digoxigenin-labelled telomeric probe. The probe was later detected by the DIG luminescent detection procedure (Roche) and exposed on a charge-coupled device camera (Las 4000, Fuji). Measurements were performed in duplicate on separate gels. The measurement repeatability, as determined by the intraclass correlation coefficient, was 0.99 (95% confidence interval, 0.817–1.0) and 0.98 (95% confidence interval, 0.81–1.0) for LTL and MTL, respectively. The repeatability of the means of two duplicates, known as the extrapolated repeatability, was 0.995 and 0.991 for LTL and MTL, respectively. The LTL/MTL ratio was calculated for each individual by dividing the LTL by the MTL value. As MTL can be considered as a proxy of TL at birth and LTL represents the current status of TL, a ratio of 1 indicates no telomere attrition throughout life, while a smaller ratio indicates lower LTL values compared to MTL and thus greater telomere attrition.

### VEGF-A protein measurement

VEGF-A protein was measured in plasma samples, using Cytokine Array I on Randox semi-automated benchtop immunoanalyser (Evidence Investigator Analyzer, Randox Laboratories Ltd., Crumlin, UK). Cytokine Array I is a high sensitivity multiplex cytokine and growth factor array, which enables simultaneous detection of 12 cytokines and growth factors in a single sample. VEGF-A plasma levels were measured in 324 French individuals from the TELARTA study.

### Genotyping

Ten VEGF-A related genetic variants (rs10761741, rs10738760, rs6921438, rs7043199, rs6993770, rs4416670, rs114694170, rs34528081, rs4782371 and rs2639990), previously identified by a GWAS, were genotyped in leukocyte DNA samples using a PCR-based KASP assay [46]. Genotyping was performed by the Laboratory of the Government Chemist (LGC Ltd., Teddington, UK) using the competitive allele-specific PCR (KASP) chemistry coupled with a Förster resonance energy transfer-based genotyping system (http://www.kbioscience.co.uk/reagents/KASP/KASP.html) and by Randox genotyping VEGF-A assay.

### Statistical analysis

Minor allele frequencies (MAF) and Hardy-Weinberg equilibrium (HWE) were calculated for ten VEGF-A related genetic variants (rs10761741, rs10738760, rs6921438, rs7043199, rs6993770, rs4416670, rs114694170, rs34528081, rs4782371 and rs2639990). The SNP rs10761741 did not follow the HW equilibrium (χ^2^ test) and was excluded from further analyses due to suspected technical error in the genotyping.

LTL was log-transformed to follow a normal distribution. The direct effects of VEGF-A related genetic variants on three phenotypes of interest (LTL, MTL and LTL/MTL) were tested using linear regression models adjusted for age and sex using the PLINK toolset under the assumption of an additive genetic model. Reference allele for all variants was the minor allele. Epistatic interactions were tested using R package CAPE.

The significance level for the direct effects of the 9 SNPs and the three tested phenotypes (LTL, MTL and LTL/MTL) was calculated as 0.05/9/3= 0.0012. For epistatic interactions, the significance level for the nine SNPs and three phenotypes was calculated as 0.05/36/3= 0.00046.

Multiple regression analyses were performed to study the association of VEGF-A plasma concentration with LTL, MTL and LTL/MTL. As VEGF-A levels were not normally distributed, a log-transformation was performed. In the models, VEGF-A levels were used as the dependent variable and they were adjusted for age, sex and statin use, because statins were significantly associated with VEGF-A levels in our population (data not presented).

### *In silico* analysis

The genomic environment of the significant SNPs was explored using Ensembl browser of the human genome (GRCh38.p12) and NCBI dbSNP.
